# Supplementary material for: Egg multivesicular bodies elicit an LC3-associated phagocytosis-like pathway to degrade paternal mitochondria after fertilization
Source: Nat Commun. 2024 Jul 8;15:5715. doi: 10.1038/s41467-024-50041-5 (PMC11231261; doi:10.1038/s41467-024-50041-5)
Supplement: Supplementary file 1 — Supplementary Information [file 41467_2024_50041_MOESM1_ESM.pdf]

Egg MVBs elicit an LC3-associated phagocytosis-like pathway to degrade paternal mitochondria after fertilization

Ben-Hur et al.

Supplementary Information

Supplementary Figures

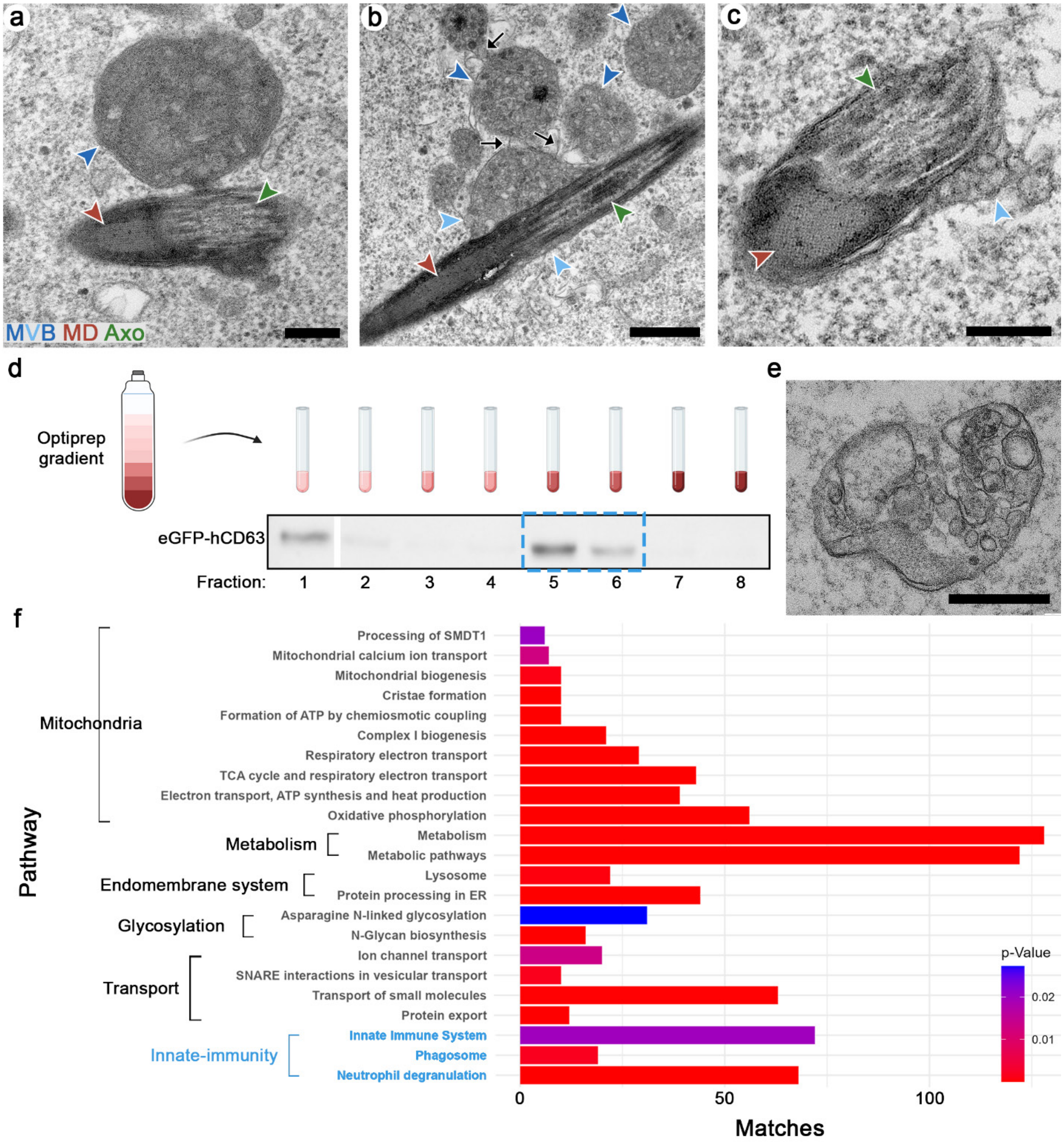

Supplementary Fig. 1: Egg-derived MVBs contain components of innate immunity with elements of phagocytosis. a-c, Electron micrographs of cross-sections through the sperm flagellar region in fertilized eggs at 10–60 min AEL. Immediately after fertilization, egg MVBs cluster around and associate with the sperm flagellum (dark and light blue arrowheads, respectively). The flagellum contains two main organelles, the mitochondrial derivative, characterized by dark

paracrystalline material (red arrowheads) and the axoneme, detected by the highly ordered 9 + 2 microtubule structure (green arrowheads). Note the protrusions that interconnect MVBs to one another, eventually forming a condensed vesicular network around the flagellum (black arrows). Scale bars in (**a,c**), 200 nm; (**b**), 500 nm. **d,** Western blot analysis of fractions obtained by OptiPrep™ density gradient isolation of MVBs from lysates prepared from eggs maternally expressing the MVB marker eGFP-hCD63. The peak of the eGFP signal appeared in fractions # 5 and 6 (band size, 52 kDa). Note that fraction #1 is the lowest density fraction, which likely contains the MVB unbound hCD63-eGFP. Uncropped blot is provided at the end of this file. **e,** A TEM micrograph of a sample from combined fractions # 5 and 6, demonstrating the enrichment of egg MVBs in these fractions. Scale bar, 0.5 µm. **f,** Pathway enrichment analysis ($P < 0.05$) of the egg MVB proteins shows enrichment of innate-immunity related factors, such as Spn88Ea (FBgn0028984), Catalase (FBgn0000261), and Ostgamma (FBgn0032015); ($P = 0.02$), and phagosome related factors, including many V-ATPase proteins, Rab 6, and Rab 7; ($P = 0.002$). Enriched pathways are presented by the number of representative genes (matches), and the corresponding P values (color scale). Pathway enrichment analysis was performed using the Flymine website. Experiments describes in (**a-c**) were repeated at least three times, while experiments in (**d-f**) were performed once. Source data are provided as a Source Data file.

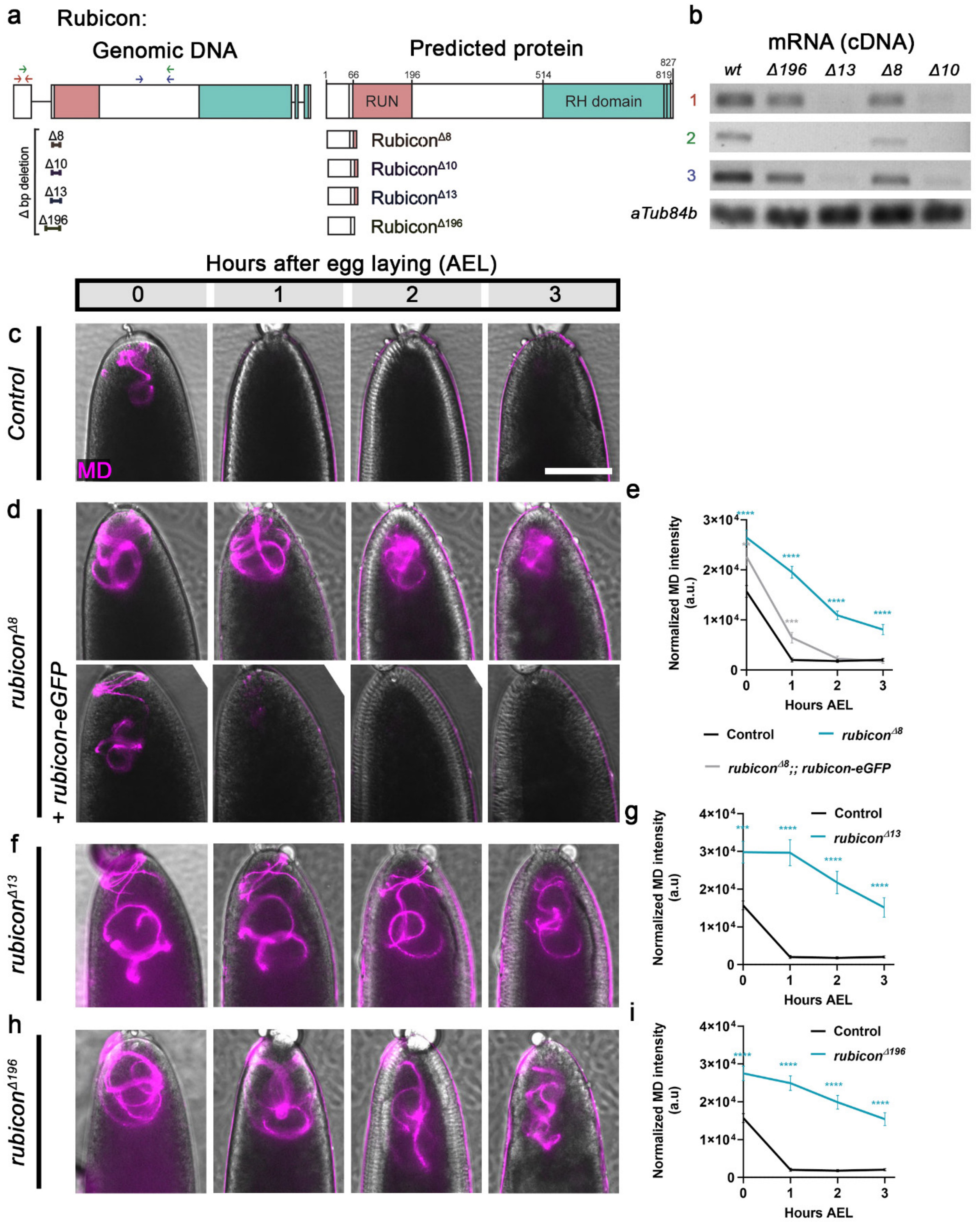

Supplementary Fig. 2: PME is significantly attenuated in *rubicon* mutant early fertilized eggs. a, Schematic representations of the *rubicon* gene and mutant alleles (genomic DNA; left),

and the matching predicted proteins (right). Exons and introns are indicated by thick and thin bars of the DNA structure, respectively. The relative locations of the RUN (pink) and Rubicon homology (RH) (turquoise) domains are indicated. Different colored bars below the DNA structure indicate the locations of the deletions in the different *rubicon* mutant alleles, which result in frameshifts and consequent premature stop codons. **b,** RT–PCR analyses of *rubicon* mRNA expression in WT and the *rubicon* mutant alleles. After reverse transcription (RT) with RNA from early fertilized eggs, PCR was performed using three sets of specific primers spanning different regions across the *rubicon* gene (colored numbers on the left correspond to the colored arrows above the genomic DNA structure in (**a**), which indicate the relative locations of the specific primer pairs). Whereas reduced mRNA levels were detected in eggs maternally mutant for all *rubicon* alleles, the $rubicon^{\Delta 13}$ maternal mutant eggs essentially displayed no *rubicon* mRNA expression, indicating that this is a *rubicon* RNA null allele. For loading control of the cDNA template, the *αTub84B* gene (CG1913) mRNA was amplified. Uncropped gel is provided at the end of this file. This experiment was performed once. **c-i,** PME is significantly attenuated in early fertilized eggs laid by females homozygous for the *rubicon* mutant alleles. **c,d,f,h,** Live imaging assays performed and presented as in Fig. 1e. Note the near complete restoration of PME kinetics in the $rubicon^{\Delta 8}$ mutant egg upon maternal expression of the *rubicon-eGFP* transgene. Scale bar, 100 µm. **e,g,i,** Quantifications of normalized red-MD fluorescence intensities [arbitrary units (a.u.)] in the early fertilized eggs respectively represented in (**c,d,f,h**). Error bars indicate SEM. The respective numbers of scored early fertilized eggs (*n*) laid by females of the control, $rubicon^{\Delta 8}$, $rubicon^{\Delta 8}$;; *rubicon-eGFP*, $rubicon^{\Delta 13}$, and $rubicon^{\Delta 196}$, are 61, 50, 47, 47, and 53. $^{**}P < 0.01$, $^{***}P < 0.001$, and $^{****}P < 0.0001$. Statistical tests in (**e**), two-way repeated measures ANOVA, followed by Dunnett's multiple comparisons test. Statistical tests in (**g,i**), two-way repeated measures ANOVA, followed by Šídák's multiple comparisons test. Source data are provided as a Source Data file.

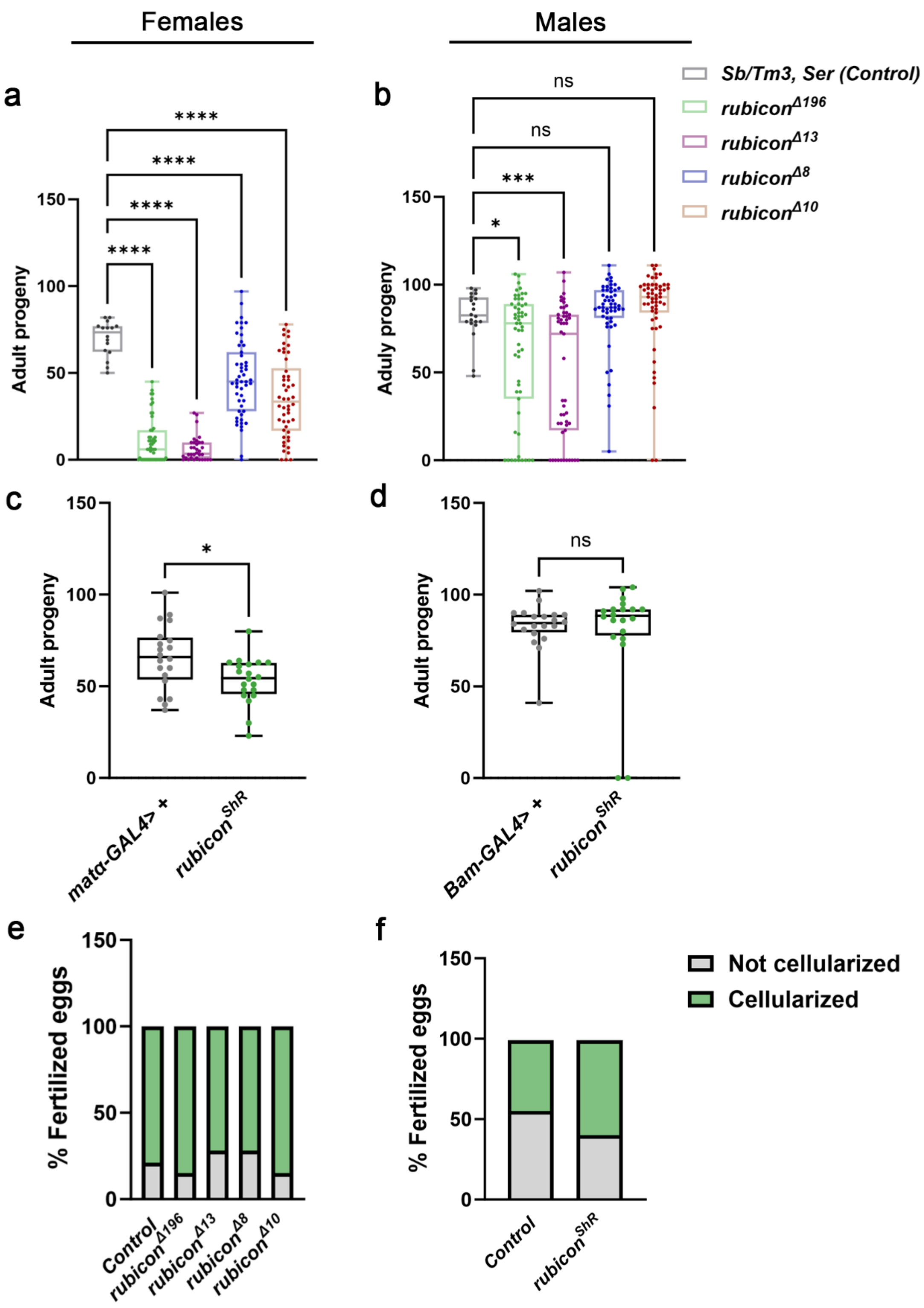

Supplementary Fig. 3: Maternal Rubicon expression is required for proper development of the fly. a-d, Fertility tests of 3-day-old virgin female (**a,c**) and male (**b,d**) flies homozygous for the four *rubicon* mutant alleles and a control genotype (**a,b**), as well as for maternal *rubicon*

knockdown female flies (**c**) and male flies with germline *rubicon* knockdown (**d**). *rubicon* deficient female and male flies were crossed with WT counterparts, and the numbers of progeny that reached the adult stage were manually counted. In accordance with the RT-PCR data (presented in Supplementary Fig. 2b), the most significant reduction in fertility was observed in females (and to a lesser extent, in males) carrying the $rubicon^{\Delta 13}$ and $rubicon^{\Delta 196}$ alleles. Note that maternal knockdown of *rubicon* also caused a significant reduction in female fertility, while germline *rubicon* knockdown had no effect on male fertility, suggesting that at least part of the effect on female fertility is due to compromised maternal *rubicon* expression, whereas the effect on male fertility is due to a possible role of Rubicon in the soma. All data points, including outliers, were presented in box plot format where the minimum is the lowest data point represented by the lower whisker bound, the maximum is the highest data point represented by the upper whisker bound, and the center is the median. The lower box bound is the median of the lower half of the dataset, while the upper box bound is the median of the upper half of the dataset. Each dot corresponds to the number of progeny obtained from a single cross. The respective numbers of crosses examined for the control, $rubicon^{\Delta 196}$, $rubicon^{\Delta 13}$, $rubicon^{\Delta 8}$, and $rubicon^{\Delta 10}$ are 16, 51, 34, 51 and 50 (**a**), and 20, 55, 51, 53 and 55 (**b**). For the control and $rubicon^{ShR}$, 20 crosses were examined for each genotype (**c,d**). $^{*}P < 0.05$, $^{***}P < 0.001$, and $^{****}P < 0.0001$. Statistical tests in (**a,b**), One-way ANOVA, followed by Dunnett's multiple comparisons test. Statistical tests in (**c,d**), Two-tailed unpaired student's t-test. **e,f,** The maternal effect on the fertility of *rubicon* deficient adult females is not due to pre-cellularization early embryonic stages. Early embryo progeny of females homozygous for the *rubicon* mutant alleles (**e**) or with maternal *rubicon* knockdown (**f**), both reached to the cellularization stage in a similar kinetics as control embryos. Embryos carrying a single copy of the *rubicon* gRNA served as control in (**e**), while embryos expressing the maternal driver alone served as control in (**f**). **e,** The respective numbers of scored embryos (*n*) laid by females of the control, $rubicon^{\Delta 196}$, $rubicon^{\Delta 13}$, $rubicon^{\Delta 8}$, and $rubicon^{\Delta 10}$ are 89, 66, 68, 75, and 82. Statistical test, two-sided Fisher's exact test. The respective *P*-values obtained by comparing each mutant to the control are: 0.4065, 0.3536, 0.3641, 0.3214. False discovery rate (FDR) *q*-value to correct for multiple-hypothesis testing is 0.4106. **f,** The respective numbers of scored embryos (*n*) laid by females of the control and $rubicon^{ShR}$ are 70 and 113. Two-sided Fisher's exact test. *P*-value = 0.0669. Source data are provided as a Source Data file.

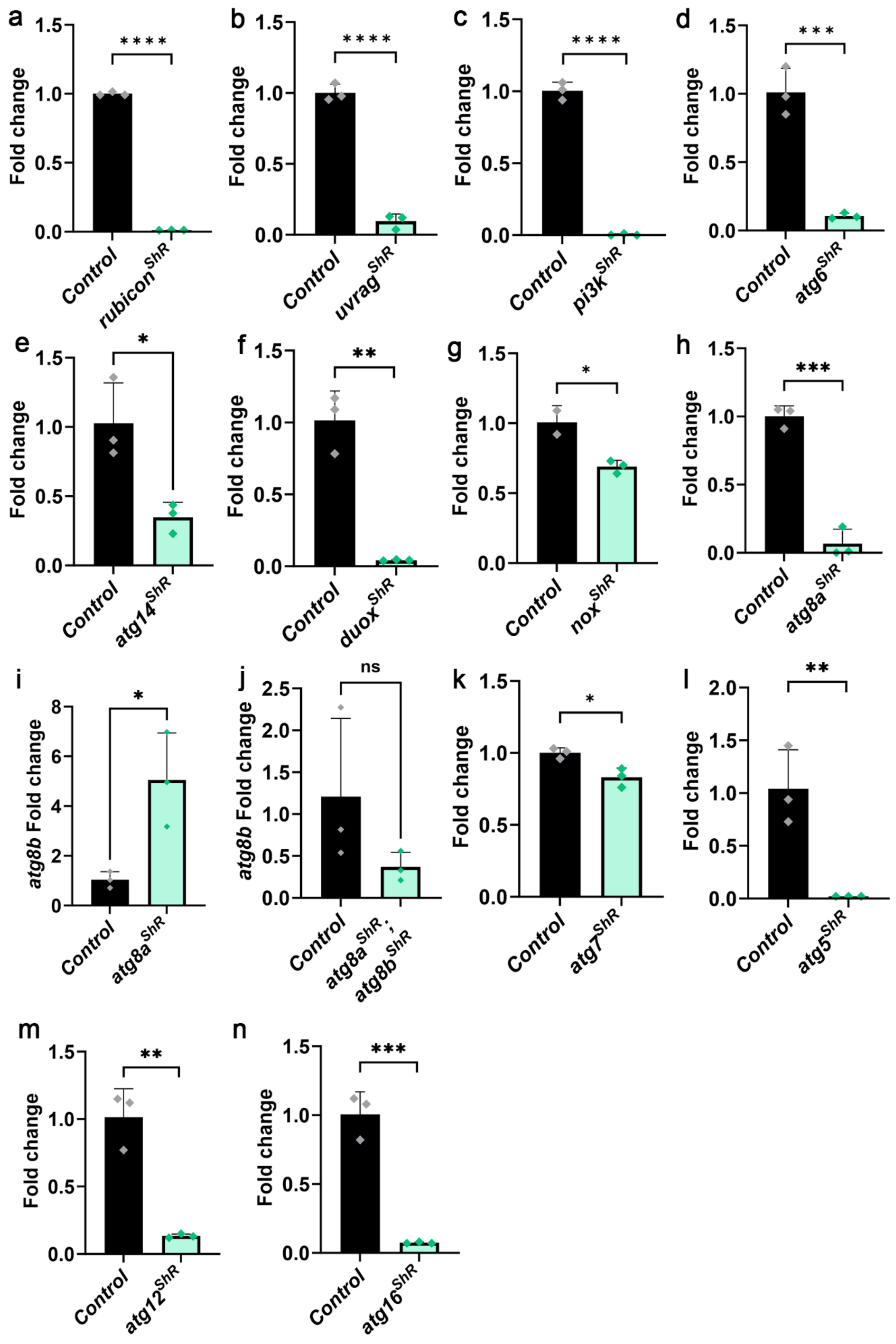
a
b
c
d
e
f
g
h
i
j
k
l
m
n
Fold change
atg8b Fold change
Control
rubicon ShR
uvrag ShR
pi3k ShR
atg6 ShR
atg14 ShR
duox ShR
nox ShR
atg8a ShR
atg8a ShR; atg8b ShR
atg7 ShR
atg5 ShR
atg12 ShR
atg16 ShR
ns

Supplementary Fig. 4: Validation of the shRNA-mediated knockdowns used in this study. a-h,k-n, Quantitative reverse transcription PCR (RT-qPCR) analyses of early fertilized eggs with maternal knockdown of *rubicon*, $P < 0.0001$, n=3 (**a**); *uvrag*, $P < 0.0001$, n=3 (**b**); *pi3k*, $P < 0.0001$, n=3 (**c**); *atg6*, $P = 0.0009$, n=3 (**d**); *atg14*, $P = 0.0195$, n=3 (**e**); *duox*, $P = 0.0012$, n=3 (**f**); *nox*, $P = 0.0221$, n=3 (**g**); *atg8a*, $P = 0.0003$, n=3 (**h**); *atg7*, $P = 0.0170$, n=3 (**k**); *atg5*, $P = 0.0088$, n=3 (**l**); *atg12*, $P = 0.0020$, n=3 (**m**); *atg16*, $P = 0.0006$, n=3 (**n**). The analyses in (**a-h,k-n**) measures the relative fold change in expression of the respective knocked down genes. **i,j,** RT-qPCR analyses of the relative fold change of *atg8b* mRNA expression in early fertilized eggs with maternal knockdown of *atg8a* alone (**i**; $^*P < 0.05$, n=3) or double knockdown of *atg8a* and *atg8b* (**j**; ns, non-significant, n=3). **a-n,** The maternal driver line (*matα-GAL4*/+) was used as control. Two-tailed unpaired student's t-test. Error bars indicate standard deviation (SD). Note that *atg8b* mRNA levels increase in early fertilized eggs with maternal *atg8a* knockdown (**i**), and that this increase is specific for *atg8b*, as it is decreases upon maternal double knockdown of *atg8a* and *atg8b* (**j**). Source data are provided as a Source Data file.

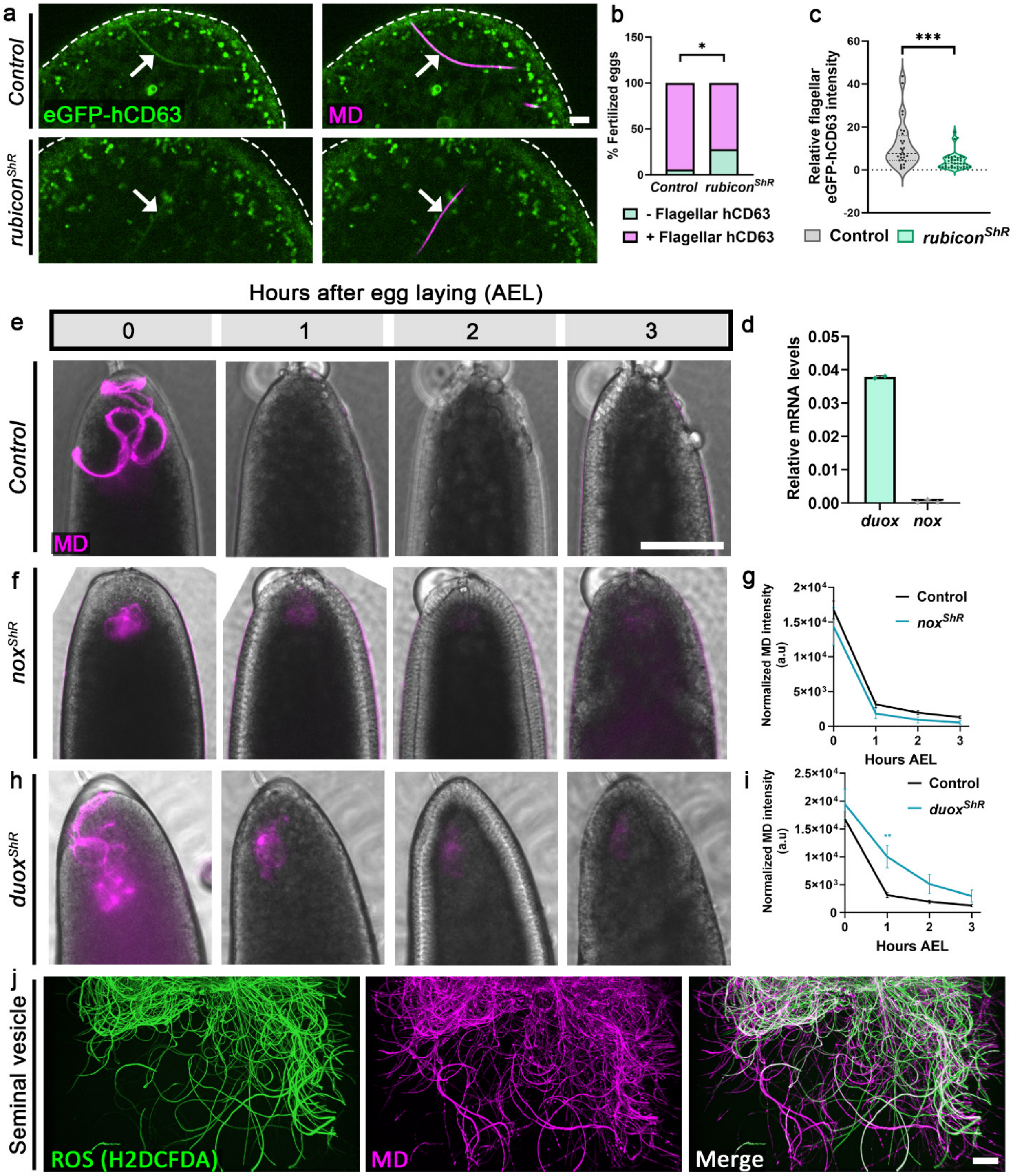

Supplementary Fig. 5: Effect of Rubicon on FVS formation and involvement of ROS in PME. **a-c,** Rubicon is required for proper biogenesis and/or recruitment of the MVBs to the sperm flagellum. Early fertilized eggs compromised for Rubicon exhibit reduced flagellar MVB association. **a,** Representative single Z-section live images of control (top) and *rubicon* knockdown

(bottom) eggs (at 0-15 min AEL) maternally expressing the MVB transgenic marker *eGFP-hCD63* (green), and fertilized by red-MD sperm cells. Note the faint eGFP-hCD63 signal emanating from the flagellum in a maternal *rubicon* knockdown egg compared with a corresponding flagellum in a control egg. Scale bar, 5 μm. **b,** Quantification of the number of early fertilized eggs (corresponding to the eggs in **a**) that display flagellar eGFP-hCD63, reveals a mild but significant reduction in the number of maternal *rubicon* downregulated eggs that display flagellar eGFP-hCD63. Two-sided Fisher's exact test. *$P < 0.05$. The respective numbers of examined fertilized eggs (n) laid by females of the control and $rubicon^{ShR}$ are 33 and 39. **c,** Quantification of the intensity of flagellar eGFP-hCD63 signal in early fertilized eggs (corresponding to the eggs in **a**), demonstrates a highly significant decrease in flagellar eGFP-hCD63 signal in the maternal *rubicon* knockdown eggs. Since the eGFP-hCD63 resides in the ILV compartment of the MVBs, the decrease in the eGFP-hCD63 signal may reflect defective MVB biogenesis and/or decreased association of the MVBs with the flagellum. Two-tailed unpaired student's t-test. ***$P < 0.001$. The respective numbers of examined fertilized eggs (n) laid by females of control and $rubicon^{ShR}$ are 28 and 40. In the violin plots, the center line represents the median of the data frequency distribution. Quartiles are represented by a dashed black line. Each dot corresponds to the relative eGFP-hCD63 mean intensity signal associated with the MD in a single fertilized egg. **d,** Duox is essentially the sole maternally expressed egg NADPH oxidase. The histogram depicts RT-qPCR analysis of relative mRNA levels of *duox* and *nox* in early fertilized WT eggs. Error bars indicate standard deviation (SD). **e,f,h,** shRNA (ShR) mediated maternal knockdown of *duox*, but not of *nox*, significantly attenuates PME. Live imaging assays performed and presented as in Fig. 1e. Scale bar, 100 μm. **g,i,** Quantifications of normalized red-MD fluorescence intensities [arbitrary units (a.u.)] in early fertilized eggs represented in (**e,f,h**). Error bars indicate SEM. The respective numbers of scored early fertilized eggs (n) laid by control, maternal nox^{ShR}, and maternal $duox^{ShR}$ females, are 68, 19, and 30. **$P < 0.01$. Two-way repeated measures ANOVA, followed by Šídák's multiple comparisons test. **j,** Mature sperm display high levels of flagellar ROS prior to fertilization. Seminal vesicles from males producing the red-MD sperm were pierced to release mature sperm and stained with the fluorescent ROS indicator H2DCFDA (green). Scale bar, 10 μm. Source data are provided as a Source Data file.

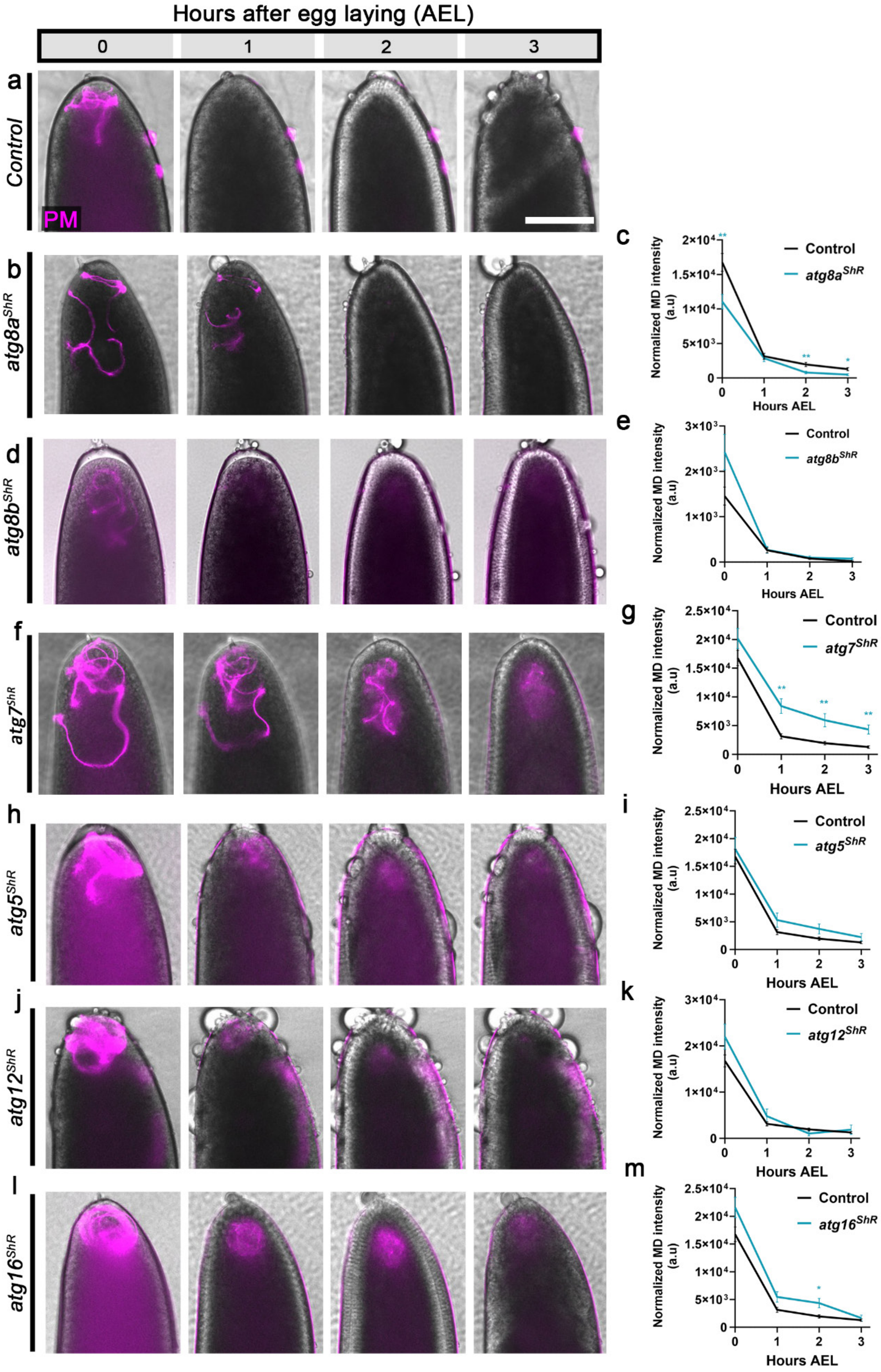

Hours after egg laying (AEL)
0
1
2
3
a
Control
PM
b
atg8a^ShR
d
atg8b^ShR
f
atg7^ShR
h
atg5^ShR
j
atg12^ShR
l
atg16^ShR
c
Normalized MD intensity (a.u)
Control
atg8a^ShR
Hours AEL
e
atg8b^ShR
g
atg7^ShR
i
atg5^ShR
k
atg12^ShR
m
atg16^ShR

Supplementary Fig. 6: Atg7, but not Atg8a/Atg8b alone or the Atg5-Atg12-Atg16 complex, is required for efficient PME. a,b,d,f,h,j,l, Live imaging assays performed and presented as in Fig. 1e. Scale bar, 100 µm. **c,e,g,i,k,m,** Quantifications of normalized red-MD fluorescence intensities [arbitrary units (a.u.)] in early fertilized eggs respectively represented in (**a,b,d,f,h,j,l**). Note the minor to no effect on the kinetics of the decaying MD transgenic fluorescent signal upon maternal shRNA-mediated downregulation of *atg8a* or *atg8b* alone (**b-e**), or of the major components of the canonical autophagy Atg8/LC3 conjugation machinery, Atg5, Atg12, and Atg16 (**h-m**). In contrast, maternal downregulation of the E1-like protein of canonical autophagy, Atg7, results in significant persistence of the MD (**f,g**). Error bars indicate SEM. The respective numbers of scored early fertilized eggs (*n*) laid by females of the control, $atg8a^{ShR}$, $atg8b^{ShR}$, $atg7^{ShR}$, $atg5^{ShR}$, $atg12^{ShR}$, and $atg16^{ShR}$ are 68, 51, 24, 33, 28, 26, and 34. $^{*}P < 0.05$ and $^{**}P < 0.01$. Two-way repeated measures ANOVA, followed by Šídák's multiple comparisons test. Source data are provided as a Source Data file.

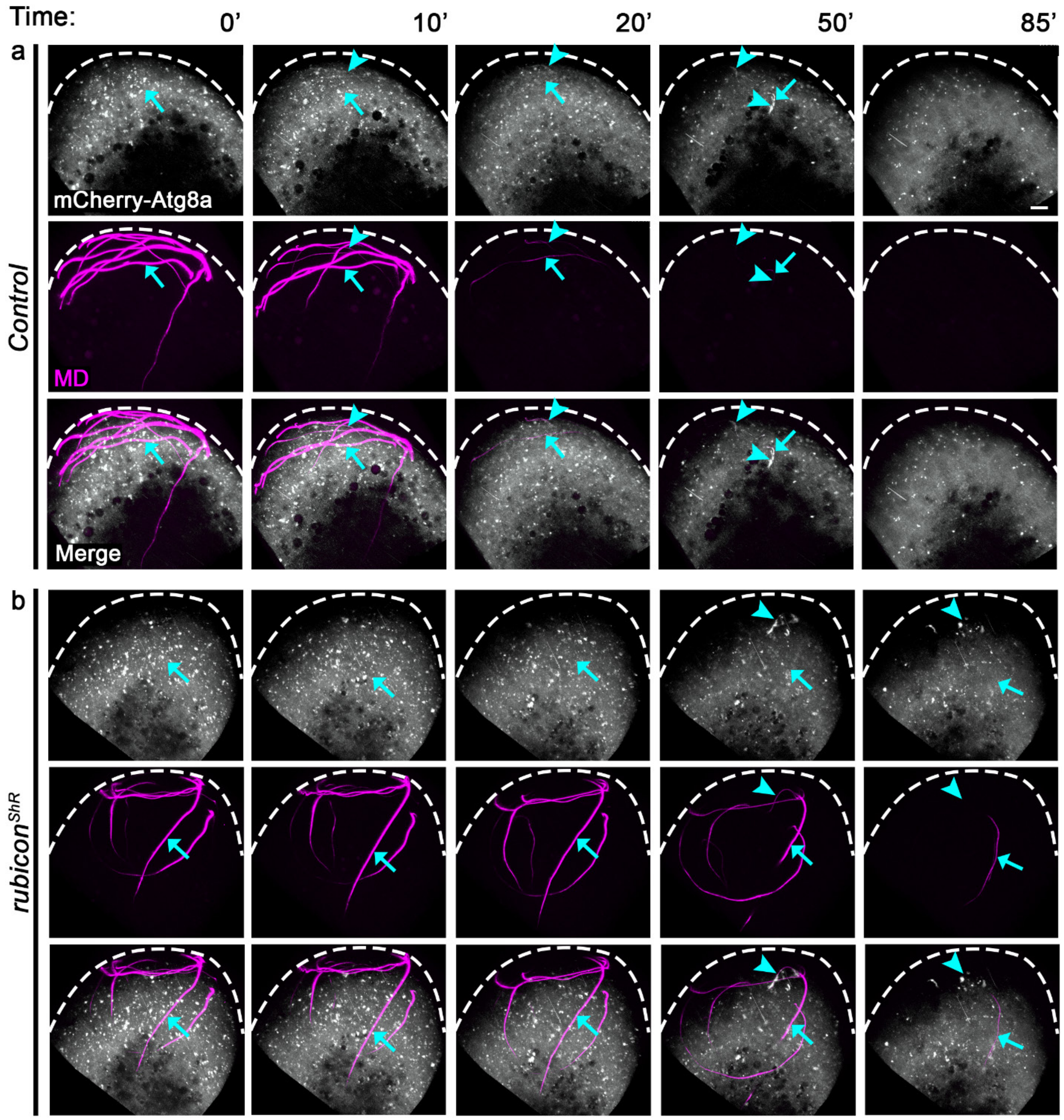

Supplementary Fig. 7: Recruitment of Atg8a to the FVS is significantly delayed and inefficient in the absence of Rubicon. a,b, Live imaging of the anterior regions of two early developing eggs fertilized by green-MD sperm (magenta) and maternally expressing an *mCherry-Atg8a* transgene (white) under the *matα-Gal4* driver (control, **a**) and, in addition, an shRNA transgene against *rubicon* (**b**). Shown are representative images from Supplementary Movie 8 (**a**) and Supplementary Movie 9 (**b**) taken at the indicated min AEL. The arrows track the same regions on the MD as the early fertilized eggs develop, while the arrowheads track mCherry-Atg8a

recruitment to the same regions on the FVS. Whereas association of mCherry-Atg8a with the FVS in an otherwise WT background is relatively fast (within 10-20 min AEL), with only a few short flagellum vesicular sheath segments displaying recruitment of mCherry-Atg8a at 40 min AEL (**a**; arrowheads), in the *rubicon* knockdown background, recruitment of the mCherry-Atg8a to the FVS is highly inefficient, such that it appears very late (50 min AEL) and only on a few short flagellum vesicular sheath segments (**b**; arrowheads). Scale bar, 10 μm.

Supplementary Tables

Supplementary Table 1: Fly strains used in this study.

Strain	Source/Publication	Identifier/stock #
P[matα4-GAL-VP16]V37	BDSC/TRiP collection	7063
rubicon^{ShR}	BDSC/TRiP collection	43276
uvrag^{ShR}	BDSC/TRiP collection	34368
pi3k59F^{ShR} (*pi3k/vps34)*	BDSC/TRiP collection	33384
atg6^{ShR}	BDSC/TRiP collection	35741
nox^{ShR}	BDSC/TRiP collection	38906
duox^{ShR}	BDSC/TRiP collection	32903
atg7^{ShR}	BDSC/TRiP collection	34369
atg8a^{ShR}	BDSC/TRiP collection	82955
atg5^{ShR}	BDSC/TRiP collection	34899
atg12^{ShR}	BDSC/TRiP collection	34675
atg16^{ShR}	BDSC/TRiP collection	58244
atg14 ^{ShR}	BDSC/TRiP collection	40858
atg8b^{ShR}	BDSC/TRiP collection	34900
nos-Cas9	BDSC	58986
nos-Cre	[1]	
dj-DJ-GFP (green-MD)	[2]	
TagRFPt-2xFYVE	[3]	
UASp-mCherry-Atg8a	[4]	
GFP-LAMP1	[5]	
Bam-Gal4	[6]	
dj-(MTS)tdTomato (red-MD)	This study	
dj-CPV (sperm plasma membrane)	This study	
UASp-eGFP-hCD63	This study	
rubicon-tdTomato (endogenous tag)	This study	
UASz-rubicon-eGFP	This study	
UASz-hCD63-tdTomato	This study	
rubicon$^{\Delta 8}$	This study	
rubicon$^{\Delta 10}$	This study	
rubicon$^{\Delta 13}$	This study	
rubicon$^{\Delta 196}$	This study	

Supplementary Table 2: Primers used in RT-qPCR experiments presented in Supplementary Fig. 4 and 5d.

Gene	Sequence
aTub84b	GATCGTGTCCTCGATTACCGC
	GGGAAGTGAATACGTGGGTAGG
rubicon	CCGAGACAGTGCGTCAATG
	CTTCCTCGCTGGGATAGTACA
uvrag	TCGCTGGAGCTAGATGATCC
	AGCTTTTCGCTGGTGTAGAAC
atg7	TCGTGGGCTGGGAGCTAAATA
	GGTTTACAGAGTTCTCAGCGAG
atg6	TGAGTGCTGCTTTTAAGCTGAA
	CACAGCGGATGGTCAATCTC
duox	TCCTATTCGGATGGGGTTTACG
	CAGTCCGGTTGAACTTTGACC
pi3k59F (pi3k/vps34)	CAACCAGGGCAGACCCTAC
	CCGAACATTGAGATGGAGGTG
nox	TCCGAATCAGCGGCAATCAAA
	ATGAGTAGCCAGCGTTGCTTC
atg16	TTGGGACATTGGCAAAAATTCCA
	CTGTCGAATCGAAATCTACGGAG
atg5	CCGGAGCCTTTCTATCTGATGA
	CCTGGTGTTCGGCGCTTAT
atg12	GCAGAGACACCAGAATCCCAG
	GTGGCGTTCAGAAGGATACAAA
atg14	CGCGCTACGAACGCCTTAT
	CCGGAGATTAGTTGCTGTAGGAG
atg8b	CATCCGCAAGCGTATCAATCT
	CGATGTCGGTGGGATCACA

Supplementary Table 3: Reagents used in this study.

Reagent	Source	Identifier
In-Fusion HD Cloning Kit	Takara-Clontech	638909
Quick-RNA Microprep Kit	Zymo Research	R1051
High-Capacity cDNA Reverse Transcription Kit	Applied Biosystems™	4368814
KAPA SYBR® FAST qPCR Master Mix Kit	KAPA Biosystems	KR0389_S-v2.17
Halocrbon oil 27	Sigma-Aldrich	H8773
Halocrbon oil 700	Sigma-Aldrich	H8898
2,7-Dichlorodihydrofluorescein diacetate (H2DCFDA)	Cayman chemical	85155
Protease inhibitor cocktail	Sigma-Aldrich	P8340
OptiPrep™ Density Gradient Medium	Sigma-Aldrich	D1556
MitoTracker™ Green FM	Thermo Fisher Scientific	M7514
D(+) Limonene	Thermo Fisher Scientific	FL/1860/07
Cocamide DEA	kind gift from Sano International (https://www.sano-international.com/	
Ethoxylated alcohol (Bio-Soft N1-7)	kind gift from Sano International (https://www.sano-international.com/	
Anti-GFP antibody	Abcam	Ab290
Biotin Anti-GFP antibody	Abcam	Ab6658
Anti-*Drosophila melanogaster* Atg8a polyclonal antibody	Creative-diagnostics	CABT-L1690
Anti-pan polyglycylated Tubulin Antibody, clone AXO 49	Sigma-Aldrich	MABS276
Anti-RFP	ROCKLAND	600-401-379
Anti-Mouse-IgG-Atto-647N	Sigma-Aldrich	50185
MA-NHS (methacrylic acid N-hydroxy succinimidyl ester)	Sigma-Aldrich	730300
Sodium acrylate	Sigma-Aldrich	408220
Acrylamide	Bio-Rad	1610140
Bisacrylamide	Bio-Rad	1610142
4-hydroxy-TEMPO	Sigma-Aldrich	176141
Tetramethylethylenediamine	Bio-Rad	1610800
Ammonium persulfate	Bio-Rad	1610700
Schneider's Drosophila Medium with L-Glutamine	Biological industries, Beit Haemek, Israel	01-150-1A
Phusion® High-Fidelity DNA Polymerase	New England Biolabs	M0530S

Supplementary Table 4: Software used in this study.

Software	Source	Identifier
The R Project for Statistical Computing	R Core Team, 2020	https://www.r-project.org/, RRID:SCR_001905
Imaris 9.5.0	Bitplane	http://www.bitplane.com/, RRID:SCR_007370
FIJI		https://imagej.net/Fiji/Downloads, RRID:SCR_002285
Arivis Vision4D		
GraphPad Prism 9.5.1	GraphPad Software, San Diego, California USA	http://www.graphpad.com
Fusion	Oxford Instruments Andor	https://andor.oxinst.com/downloads/view/fusion-user-guide
StepOnePlus ™ Real-Time PCR System	Applied Biosystems ™ 4376600	https://www.thermofisher.com/order/catalog/product/4376600
Ilastik AutoContext pixel classifier 1.4.0		https://www.ilastik.org/documentation/autocontext/autocontext

Uncropped scans of all blots and gels

Supplementary Fig. 1d

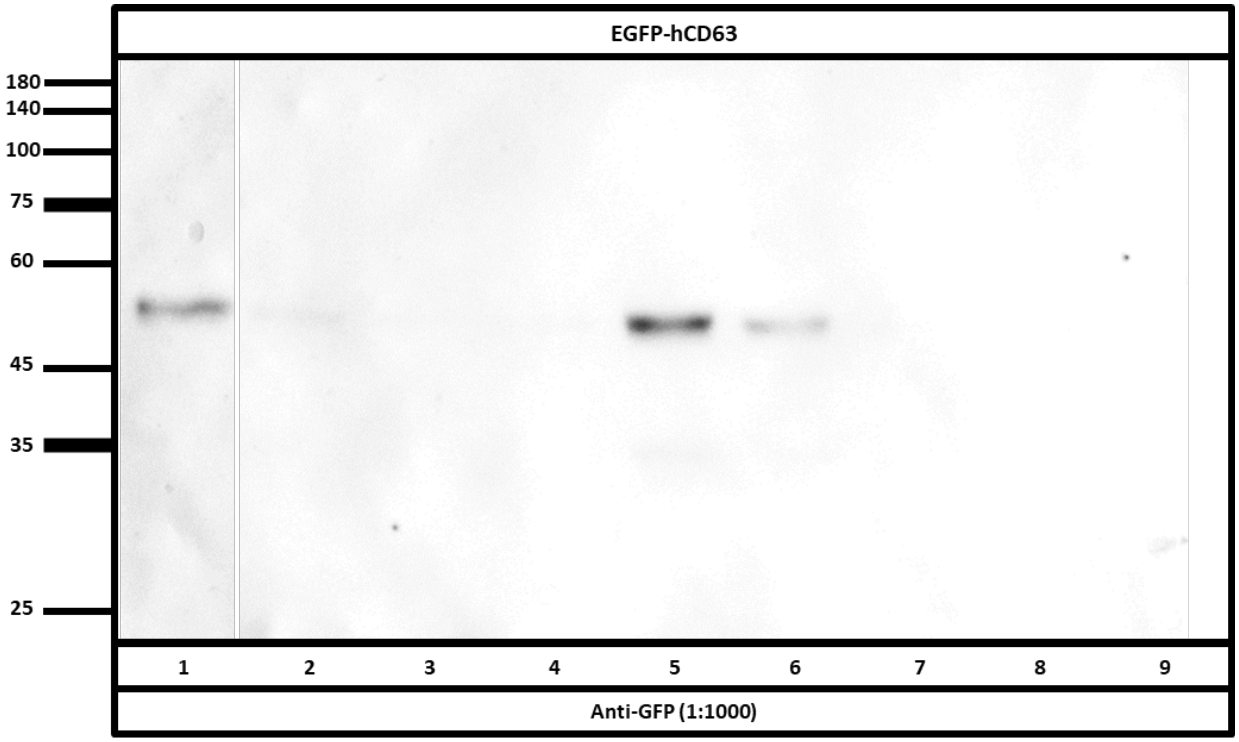

Supplementary Fig. 2b

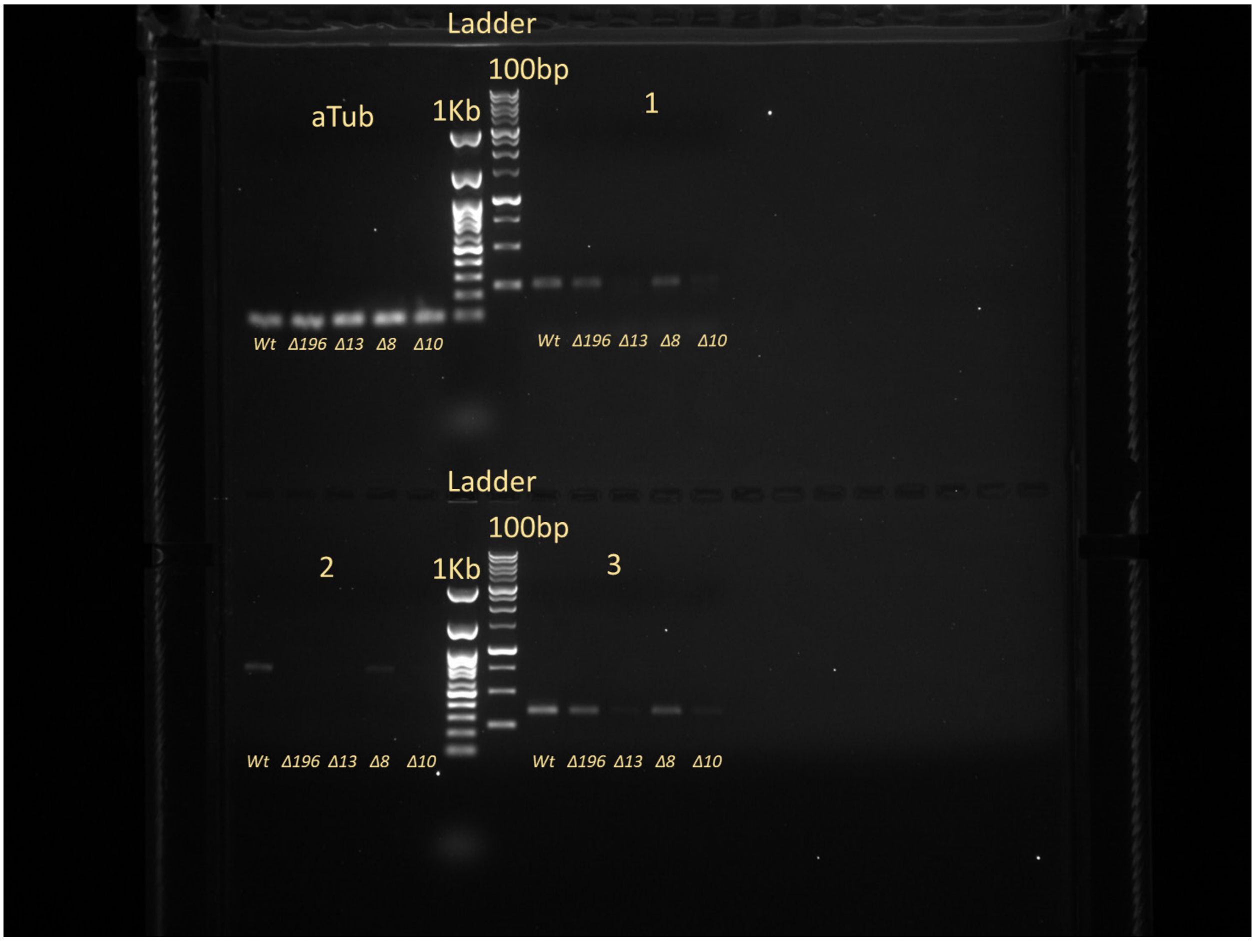

Supplementary References

1. Takeo, S., Swanson, S. K., Nandanan, K., Nakai, Y., Aigaki, T., Washburn, M. P., Florens, L. & Hawley, R. S. Shaggy/glycogen synthase kinase 3β and phosphorylation of Sarah/regulator of calcineurin are essential for completion of Drosophila female meiosis. *Proc Natl Acad Sci U S A* **109**, 6382–6389 (2012).
2. Bazinet, C. & Rollins, J. E. Rickettsia-like mitochondrial motility in Drosophila spermiogenesis. *Evol Dev* **5**, 379–385 (2003).
3. Liu, G., Sanghavi, P., Bollinger, K. E., Perry, L., Marshall, B., Roon, P., Tanaka, T., Nakamura, A. & Gonsalvez, G. B. Efficient endocytic uptake and maturation in drosophila oocytes requires Dynamitin/p50. *Genetics* **201**, 631–649 (2015).
4. Rusten, T. E. *et al.* ESCRTs and Fab1 Regulate Distinct Steps of Autophagy. *Current Biology* **17**, 1817–1825 (2007).
5. Akbar, M. A., Ray, S. & Krämer, H. The SM protein Car/Vps33A regulates SNARE-mediated trafficking to lysosomes and lysosome-related organelles. *Mol Biol Cell* **20**, 1705–1714 (2009).
6. Aram, L., Braun, T., Braverman, C., Kaplan, Y., Ravid, L., Levin-Zaidman, S. & Arama, E. A Krebs Cycle Component Limits Caspase Activation Rate through Mitochondrial Surface Restriction of CRL Activation. *Dev Cell* **37**, 15–33 (2016).
