# Supplementary material for: Egg multivesicular bodies elicit an LC3-associated phagocytosis-like pathway to degrade paternal mitochondria after fertilization
Source: Nat Commun. 2024 Jul 8;15:5715. doi: 10.1038/s41467-024-50041-5 (PMC11231261; doi:10.1038/s41467-024-50041-5)
Supplement: Supplementary file 3 — Description of Additional Supplementary Files [file 41467_2024_50041_MOESM3_ESM.pdf]

Description of additional supplementary files

Supplementary Data 1

Protein profiling of the egg MVBs. Sheet 1: Total proteins identified by the MS analysis in the MVB and lysate (control) fractions. Sheet 2: MVB enriched proteins considered for the pathway enrichment analysis. The list contains all the proteins for which the fold change ratio (log2 of MVB PSM-log2 of lysate PSM) is above 1. In addition, proteins with less than 10 PSMs were discarded

Supplementary Movie 1

Live imaging of PME after fertilization in an early *Drosophila* fertilized egg. A time-lapse confocal microscopy movie of the anterior region of a WT egg fertilized by red-MD sperm (magenta; *dj-(MTS)tdTomato*). The early fertilized egg was imaged for 43 min AEL. Note the MD pieces that bud off from the flagellum as PME advances (arrow). Scale bar, 10 µm.

Supplementary Movie 2

Live imaging of PME in an early fertilized egg maternally expressing the *rubicon-eGFP transgene*. A time-lapse confocal microscopy movie of the anterior region of a WT egg maternally expressing the *UASz-rubicon-eGFP* transgenic construct (green) and fertilized by red-MD sperm (magenta; *dj-(MTS)tdTomato*). Rubicon positive MVBs engage and densely coat the sperm flagellum, forming FVS that enwrap large flagellar segments withing which the MD is degraded. The early fertilized egg was imaged for 45 min AEL. Scale bar, 10 µm.

Supplementary Movie 3

Expansion microscopy (ExM) imaging of the flagellar region in early fertilized egg expressing Rubicon-tdTomato. Superresolution imaging of flagellar regions in eggs maternally expressing the *UASz-rubicontdTomato* transgenic construct (magenta), fertilized by green-MD sperm (green; *dj-DJGFP*), and prepared for ExM. The images feature large MD segments that are readily enwrapped within Rubicon positive FVS, as well as large Rubicon positive vesicles that contain MD fragments and bud off from the flagellum. Note that the video begins with a non-computed 3D projection and is followed by a 3D computer rendering of segmented surfaces. Multiple nonspecific green puncta scattered throughout the non-computed 3D projection may correspond to endogenously biotinylated proteins (as staining of the MD involved an anti-GFP antibody conjugated to Biotin, followed by a fluorescently tagged streptavidin). Scale bar, 5 µm.

Supplementary Movie 4

Live imaging of sperm plasma membrane breakdown in an early fertilized egg maternally expressing the rubicon-tdTomato transgene. A time-lapse confocal microscopy movie of the anterior region of an egg maternally expressing the *UASz-rubicontdTomato* transgenic construct (magenta) and fertilized by a sperm cell expressing the *dj-CD8- Venus* transgenic construct of the plasma membrane (green). Note that complete breakdown of the sperm plasma membrane occurs within 8-10 min AEL. Scale bar, 3 µm.

Supplementary Movie 5

Live imaging of PME in an early fertilized egg maternally expressing a PtdIns(3)P reporter. A time-lapse confocal microscopy movie of the anterior region of a WT egg maternally expressing the PtdIns(3)P transgenic reporter construct *UASp-TagRFPt-2xFYVE* (magenta) and fertilized by green-MD sperm (green; dj-DJGFP). The early fertilized egg was imaged for 15 min AEL. Note the association of PtdIns(3)P on the FVS (arrow), indicative of PI3KC3 activity. Scale bar, 10 µm.

Supplementary Movie 6

Live imaging of PME in an early fertilized egg maternally expressing both Rubicon-GFP and a PtdIns(3)P reporter. A time-lapse confocal microscopy movie of the anterior region of an early fertilized egg maternally expressing the *UASz-rubicon-eGFP* transgenic construct (green) and the PtdIns(3)P transgenic reporter construct *UASp-TagRFPt2xFYVE* (magenta), and fertilized by WT sperm. Both Rubicon and PtdIns(3)P associate with the sperm flagellum already at time zero AEL. Scale bar, 5 µm.

Supplementary Movie 7

Live imaging of PME in an early fertilized egg maternally expressing both the Rubicon-GFP and an mCherry-Atg8a. A time-lapse confocal microscopy movie of the anterior region of a WT egg maternally expressing the *UASz-rubicon-eGFP* (green) and the *UASp-mCherry-atg8a* (magenta) transgenic constructs, and fertilized by WT sperm. The early fertilized egg was imaged for 60 min AEL. Note the 6-12 min lag between the formation of Rubicon-eGFP positive FVS and the consecutive recruitment of mCherry-Atg8a. Scale bar, 5 µm

Supplementary Movie 8

Live imaging of PME in an early fertilized egg maternally expressing an mCherry-Atg8a. A time-lapse confocal microscopy movie of the anterior region of a WT egg maternally expressing the *UASp-mCherry-atg8a* transgenic construct (white) and fertilized by green-MD sperm (magenta; *dj-DJ-GFP*). The early fertilized egg was imaged for 90 min AEL. Note that mCherry-Atg8a is recruited to large MD segments within 10 minutes after egg laying (arrow). Scale bar, 10 µm.

Supplementary Movie 9

Live imaging of PME in an early fertilized egg compromised for rubicon and maternally expressing an mCherry-Atg8a. A time-lapse confocal microscopy movie of the anterior region of an egg maternally expressing *rubiconShR* and *UASp-mCherry-atg8a* transgenic construct (white) and fertilized by green-MD sperm (magenta; *dj-DJ-GFP*). The early fertilized egg was imaged for 3 hours AEL. Note that in the few events when mCherry-Atg8a is recruited to the FVS, it occurs only after 50 min AEL and on short flagellum vesicular sheath segments (arrow), while most of the flagellum is not associated with mCherry-Atg8a. Scale bar, 10 µm.

Supplementary Movie 10

Live imaging of PME in an early fertilized egg maternally expressing a transgenic lysosomal reporter. A time-lapse confocal microscopy movie of the anterior region of an early fertilized egg maternally

expressing the lysosomal transgenic reporter construct *tubGFP-LAMP1* (magenta), and fertilized by a red-MD sperm cell (green; *dj-(MTS)tdTomato*). Note the co-localization of the lysosomal reporter with degrading MD fragments derived from the flagellum. Scale bar, 10 µm.
