# Supplementary material for: Egg multivesicular bodies elicit an LC3-associated phagocytosis-like pathway to degrade paternal mitochondria after fertilization
Source: Nat Commun. 2024 Jul 8;15:5715. doi: 10.1038/s41467-024-50041-5 (PMC11231261; doi:10.1038/s41467-024-50041-5)
Supplement: Supplementary file 15 — Reporting Summary [file 41467_2024_50041_MOESM15_ESM.pdf]

nature portfolio

Corresponding author(s): Eli Arama

Last updated by author(s): 2024/06/03

Reporting Summary

Nature Portfolio wishes to improve the reproducibility of the work that we publish. This form provides structure for consistency and transparency in reporting. For further information on Nature Portfolio policies, see our Editorial Policies and the Editorial Policy Checklist.

Statistics

For all statistical analyses, confirm that the following items are present in the figure legend, table legend, main text, or Methods section.

n/a	Confirmed	
☐	☒	The exact sample size (*n*) for each experimental group/condition, given as a discrete number and unit of measurement
☐	☒	A statement on whether measurements were taken from distinct samples or whether the same sample was measured repeatedly
☐	☒	The statistical test(s) used AND whether they are one- or two-sided<br>*Only common tests should be described solely by name; describe more complex techniques in the Methods section.*
☒	☐	A description of all covariates tested
☒	☐	A description of any assumptions or corrections, such as tests of normality and adjustment for multiple comparisons
☐	☒	A full description of the statistical parameters including central tendency (e.g. means) or other basic estimates (e.g. regression coefficient) AND variation (e.g. standard deviation) or associated estimates of uncertainty (e.g. confidence intervals)
☐	☒	For null hypothesis testing, the test statistic (e.g. F, t, r) with confidence intervals, effect sizes, degrees of freedom and P value noted<br>*Give P values as exact values whenever suitable.*
☒	☐	For Bayesian analysis, information on the choice of priors and Markov chain Monte Carlo settings
☒	☐	For hierarchical and complex designs, identification of the appropriate level for tests and full reporting of outcomes
☐	☒	Estimates of effect sizes (e.g. Cohen's *d*, Pearson's *r*), indicating how they were calculated

Our web collection on statistics for biologists contains articles on many of the points above.

Software and code

Policy information about availability of computer code

Data collection: No Software was used for data collection

Data analysis	All graphs and statistical analyses in this manuscript were generated using the GraphPad Prism software version 9.5.1 for Windows (GraphPad Software, San Diego, California USA, www.graphpad.com), except for the ones that evaluate the co-localization of Rubicon vesicles with hCD63, which was done using the colocr R package (R Core Team, 2020 https://www.r-project.org/, RRID:SCR_001905).<br><br>MIP (Max-Intensity-Projection) processing of images was performed using Imaris 9.5.0 (Bitplane, http://www.bitplane.com/, RRID:SCR_007370).<br><br>When needed, images were deconvolved with the internal Andor Fusion (version 2.4) deconvolution application.<br><br>Pathway enrichment analysis (Supplementary Fig. 1f) was performed using the Flymine platform (https://www.flymine.org/flymine).<br><br>The costume code used to quantify PME kinetics was implemented as a Fiji 125 macro (Windows-64 version), using the Ilastik AutoContext pixel classifier (1.4.0) for embryo segmentation. The code is deposited into Github repository [.github.com/WIS-MICC-CellObservatory/PME_Kinetics].<br><br>Analyses in figures 4d, 5c 7e and Supplementary Fig. 5c were performed using Arivis Vision4D. All Arivis pipelines used in this study are deposited in GitHub [github.com/WIS-MICC-CellObservatory/Atg8a-and-Axoneme-analysis/tree/main].<br><br>Real time PCR analysis was performed using StepOnePlus™ Real-Time PCR System (Applied Biosystems™, 4376600).

For manuscripts utilizing custom algorithms or software that are central to the research but not yet described in published literature, software must be made available to editors and reviewers. We strongly encourage code deposition in a community repository (e.g. GitHub). See the Nature Portfolio guidelines for submitting code & software for further information.

Data

Policy information about availability of data

All manuscripts must include a data availability statement. This statement should provide the following information, where applicable:

- Accession codes, unique identifiers, or web links for publicly available datasets
- A description of any restrictions on data availability
- For clinical datasets or third party data, please ensure that the statement adheres to our policy

The authors declare that the data supporting the findings of this study are available within the paper and its supplementary information files, and that all additional data are publicly available.
The MVB proteomics data generated in this study have been deposited in the MassIVE database under accession code MSV000093306 [massive.ucsd.edu]. Arivis pipelines generated in this study are deposited into Github repository [github.com/WIS-MICC-CellObservatory/Atg8a-and-Axoneme-analysis/tree/main]. Source data are provided with this paper.

Research involving human participants, their data, or biological material

Policy information about studies with human participants or human data. See also policy information about sex, gender (identity/presentation), and sexual orientation and race, ethnicity and racism.

Reporting on sex and gender	*Use the terms sex (biological attribute) and gender (shaped by social and cultural circumstances) carefully in order to avoid confusing both terms. Indicate if findings apply to only one sex or gender; describe whether sex and gender were considered in study design; whether sex and/or gender was determined based on self-reporting or assigned and methods used. Provide in the source data disaggregated sex and gender data, where this information has been collected, and if consent has been obtained for sharing of individual-level data; provide overall numbers in this Reporting Summary. Please state if this information has not been collected. Report sex- and gender-based analyses where performed, justify reasons for lack of sex- and gender-based analysis.*
Reporting on race, ethnicity, or other socially relevant groupings	*Please specify the socially constructed or socially relevant categorization variable(s) used in your manuscript and explain why they were used. Please note that such variables should not be used as proxies for other socially constructed/relevant variables (for example, race or ethnicity should not be used as a proxy for socioeconomic status). Provide clear definitions of the relevant terms used, how they were provided (by the participants/respondents, the researchers, or third parties), and the method(s) used to classify people into the different categories (e.g. self-report, census or administrative data, social media data, etc.) Please provide details about how you controlled for confounding variables in your analyses.*
Population characteristics	*Describe the covariate-relevant population characteristics of the human research participants (e.g. age, genotypic information, past and current diagnosis and treatment categories). If you filled out the behavioural & social sciences study design questions and have nothing to add here, write "See above."*
Recruitment	*Describe how participants were recruited. Outline any potential self-selection bias or other biases that may be present and how these are likely to impact results.*
Ethics oversight	*Identify the organization(s) that approved the study protocol.*

Note that full information on the approval of the study protocol must also be provided in the manuscript.

Field-specific reporting

Please select the one below that is the best fit for your research. If you are not sure, read the appropriate sections before making your selection.

☒ Life sciences ☐ Behavioural & social sciences ☐ Ecological, evolutionary & environmental sciences

For a reference copy of the document with all sections, see nature.com/documents/nr-reporting-summary-flat.pdf

Life sciences study design

All studies must disclose on these points even when the disclosure is negative.

Sample size	No statistical method was used to predetermine sample size. Sample size was chosen according to standard practices in the laboratory and general area of study. For the PME kinetics assay, more than 20 early fertilized eggs were used. Please refer to the following study: Politi et al., Developmental Cell, 2014.
Data exclusions	Proteomic analysis (Supplementary Data 1 and Supplementary Fig. 1): To reduce background noise, proteins with the lowest abundance (PSM < 10) were discarded from the proteomic analysis (from both treatment and control).<br>Atg8a recruitment experiment (Fig. 7e): To save computation time and avoid background signal, 40% of the smallest Atg8a objects were filtered out from each image, such that only 60% of the total Atg8a volume was further analyzed. Using a subset of the data, we verified that the same spatial distribution of the Atg8a objects is obtained when analyzing all the objects or only the larger (60%) objects as described.
Replication	All this information is included in the "Data visualization, statistics, and reproducibility" section in the Methods
Randomization	Not necessary or applicable for our approaches as all of our data were well-controlled wild type vs mutant/RNAi knockdown comparisons. Randomization is usually utilized in scientific studies to prevent bias towards certain experimental groups. However, our study is inherently biased towards either wild type embryos or mutant embryos.
Blinding	Also not necessary or applicable for our approaches as all of our data were well-controlled wild type vs mutant/RNAi knockdown comparisons. Additionally, our mutant phenotypes are highly distinct, obviating the requirement for blinding, such that each of the co-authors who looked at embryo samples could easily score the mutant vs the wild-type.

Reporting for specific materials, systems and methods

We require information from authors about some types of materials, experimental systems and methods used in many studies. Here, indicate whether each material, system or method listed is relevant to your study. If you are not sure if a list item applies to your research, read the appropriate section before selecting a response.

Materials & experimental systems

n/a	Involved in the study
☐	☒ Antibodies
☒	☐ Eukaryotic cell lines
☒	☐ Palaeontology and archaeology
☐	☒ Animals and other organisms
☒	☐ Clinical data
☒	☐ Dual use research of concern
☒	☐ Plants

Methods

n/a	Involved in the study
☒	☐ ChIP-seq
☒	☐ Flow cytometry
☒	☐ MRI-based neuroimaging

Antibodies

Antibodies used	Anti-GFP antibody Abcam Ab290 (diluted 1:1000<br>Biotin Anti-GFP antibody Abcam Ab6658 (diluted 1:100)<br>Anti-RFP ROCKLAND 600-401-379 (diluted 1:100)<br>Anti-Drosophila melanogaster Atg8a polyclonal antibody Creative-diagnostics CABT-L1690 (diluted 1:100)<br>Anti-pan polyglycylated Tubulin Antibody, clone AXO 49 Sigma-Aldrich MABS276 (diluted 1:5000)
Validation	Anti-GFP antibody Abcam Ab290: This antibody was referenced in at least 3088 studies. For the specific studies please refer to the following link: https://www.abcam.com/products/primary-antibodies/gfp-antibody-ab290.html. Species independent.<br>Biotin Anti-GFP antibody Abcam Ab6658: This antibody was referenced in at least 115 studies. For the specific studies please refer to the following link: https://www.abcam.com/products/primary-antibodies/biotin-gfp-antibody-ab6658.html. Species independent.<br>Anti-RFP ROCKLAND 600-401-379: This antibody was referenced in at least 928 studies. For the specific studies please refer to the following link: https://www.rockland.com/categories/primary-antibodies/rfp-antibody-pre-adsorbed-600-401-379/. Species independent.<br>Anti-Drosophila melanogaster Atg8a polyclonal antibody Creative-diagnostics CABT-L1690. was validated by immunofluorescence in atg8a knockdown embryos (see Fig. 7).

Anti-pan polyglycylated Tubulin Antibody, clone AXO 49 Sigma-Aldrich MABS276. This antibody was extensively characterized and validated in both mammals and Drosophila (Bre et al., 1996, J Cell Sci; Bre et al., 1998, Mol Biol Cell; Arama et al., 2007, PLOS biol.).

Animals and other research organisms

Policy information about studies involving animals; ARRIVE guidelines recommended for reporting animal research, and Sex and Gender in Research

Laboratory animals	Drosophila melanogaster: This organism does not require Institutional Animal Care and Use Committee (IACUC) oversight. The mutant and transgenic strains used in the study are listed under "Fly strains used in this study" in the Supplementary Table 1.
Wild animals	This study did not involve wild animals.
Reporting on sex	Mixed sex animals were used and data were not analyzed deferentially with respect to sex.
Field-collected samples	This study did not involve samples collected from field.
Ethics oversight	*Identify the organization(s) that approved or provided guidance on the study protocol, OR state that no ethical approval or guidance was required and explain why not.*

Note that full information on the approval of the study protocol must also be provided in the manuscript.
